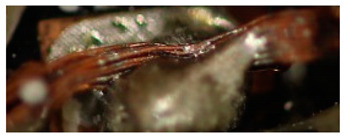# Soldering Electronics to Smart Textiles by Pulsed Nd:YAG Laser

**DOI:** 10.3390/ma13112429

**Published:** 2020-05-26

**Authors:** Sebastian Micus, Michael Haupt, Götz T. Gresser

**Affiliations:** 1 German Institutes for Textile and Fiber Research Denkendorf (DITF), 73770 Denkendorf, Germany; michael.haupt@ditf.de (M.H.); goetz.gresser@ditf.de (G.T.G.); 2 Institute for Textile and Fiber Technologies (ITFT), University of Stuttgart, 70569 Stuttgart, Germany

**Keywords:** smart textiles, wearables, laser, soldering, bonding, integration of electronics

## Abstract

Experts attest the smart textiles market will have high growth potential during the next ten years. Laser soldering is considered to be a good contacting method because it is a contactless process. For this reason, it is intended to investigate the contacting process of printed circuit boards (PCB) to isolated conductive textile strips by means of a ytterbium-doped fiber laser (1064 nm). During the investigation, the copper strands in the textile tape were stripped by the laser and soldered to the PCB without any transport of the textile. Therefore, we investigated different sets of parameters by means of a design of experiment (DoE) for different types of solder pastes. Finally, the joinings were electrically analyzed using a contact resistance test, optically with a REM examination, and mechanically using a peeling test.

## 1. Introduction

### 1.1. Smart Textiles Definition and Classification

Smart textiles are hybrid products with a functional add-on. They consist of intelligent or functionalized textiles and have an integrated sensor and/or actuator system [1]. This functional plus is distinguished between pneumatic-, light- and sound-textiles, climate control, and conductive e-textiles. They can also be used in passive, active, and very smart textiles [2].

**Figure 1:**
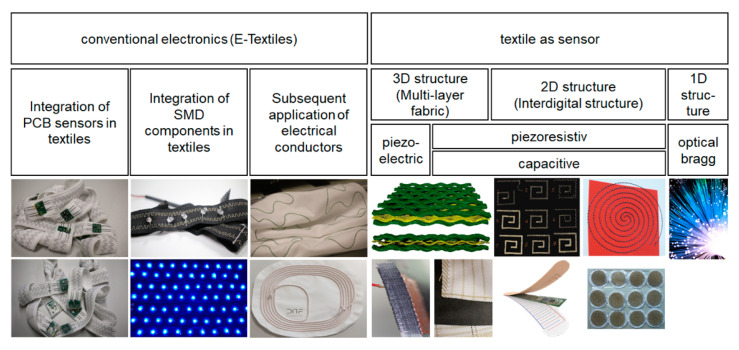
Classification of different smart textiles: E-textiles, where conventional electronics are integrated into textile and the textile structures which works as sensors.

Smart textiles can be divided into two categories (cf. Figure 1):

(1) E-textiles–these are textiles with conventional electronics where the textile works as carrier material, and;
(2) Textiles in which the textile structure itself acts as a sensor

The former category offers many more possibilities and functional principles. Furthermore, textile sensors are not able to measure as accurately as conventional electronics. They have a much stronger miniaturization, but the integration leads to local thickening. Conventional electronics added to textiles can be divided in terms of functionality. There are integrated cables for heating or charging inductively, followed by electrically-contacted SMD parts and sensors on PCBs. PCB sensors offer high accuracy at a small size. 

Textile sensors have the advantage of structural integration, though a limited number of physical principles, which are optical, capacitive, resistive, and piezoelectric [3,4,5]. Structures are mostly in the millimeter range. 

Current studies anticipate a high growth of the smart textile market in the upcoming years. Including the existing medical, military, and occupational safety markets, it will also be spread to the fashion and sports market [6]. However, at the moment there is no actual market breakthrough as there are a few factors hindering the process, i.e., a high share of manual tasks, high prices due to the manual processes, and the washability of the electronic textiles. Hence, there is currently a lot of research on automatable techniques assembling PCB to textiles. 

In this paper, our main focus is on isolated wires, because they have low resistance and the insulation provides protection against electric shocks. 

The production process of e-textiles with PCBs and insulated wires consists of three steps. At first, the wires are automatically stripped by laser. Then, the main contacting process starts [7]. In order to protect the electronics from environmental influences, the components are encapsulated in an injection molding machine [8]. The first two steps are investigated in this paper.

### 1.2. Methods for Integration

A number of studies have already been conducted on the rational contacting of electronics on textiles.

#### 1.2.1. Sewing and Embroidery

Linz et al. [9] developed an embroidery process for the efficient application of LEDs to textile surfaces requires prefabricated electronic substrates in the form of preassembled flexible electronic boards in sequin. The sequins are connected and contacted with non-insulated silver-coated conductive yarns. These yarns must survive the embroidery processes without damage, which limits the choice of material. However, these uninsulated conductors are sensitive to corrosion in washing processes, and subsequent insulation damages the textile feel. 

Afterward, Linz et al. [10] used interposers for contacting, which made the contacting process much more efficient. However, the problem of contacts with a high resistance still exists.

Therefore, Afroj et al. [11] investigated a highly-conductive, scalable, and machine-washable graphene-based yarn for embroidery. Since the surfaces to be embroidered must be tensioned during the embroidery process, embroidering elastic structures is very difficult and time-consuming.

It has also been shown that the contact resistance of a sewn or embroidered connection deteriorates over time because the textile conductors relax [11]. For this reason, the connections must be elaborately reinforced by an additional process step, e.g., gluing or soldering [9].

#### 1.2.2. Bonding

In Stoppa et al. [12], tissues with non-insulated conductors were fabricated at defined distances. In this work, µ-LEDs (200 µm wide) were placed on them and sprayed (jetted) with adhesive for fixation. However, their contact resistances increased significantly after the first washing cycles [13].

There are approaches to the production of flexible interposers. Meander-shaped copper tracks in elastic foils made of, e.g., polyurethane and PDMS are used. These can also be stretched [14,15]. Investigations with copper meanders in an elastomer (PDMS) showed that only 200 to 400 cycles can be endured at a cyclic elongation of 20% [13].

#### 1.2.3. Crimping

Simon et al. [16] described how modules with SMD components can be connected to textile-integrated conductors via crimp contacts. However, the modules with 2 × 2 cm² are still too large and have to be specially manufactured. This method only becomes interesting for very large quantities of several million pieces.

Vanfleteren et al. [17], Simon et al. [18], and Vicard [19] worked on developing interposers with form-fit connections. The interposers are pressed between two parallel conductors until the conductors are fixed to recesses on each side of the interposer. The interposers are contacted with the conductors via these recesses. 

Neudeck et al. [20] worked on interposers containing electrical components inside which are connected to the contact via bonding wires. However, the interposer must be manufactured separately for each SMD component or chip. Due to the interposer, the space required for small components such as a temperature sensor is approximately doubled, thus requiring large contact points. In addition, only two components can be contacted to one conductive yarn.

#### 1.2.4. Soldering

Molla et al. [21] integrated LEDs into textile structures by soldering conductive uninsulated yarns. The LEDs could be soldered to the exposed conductors, but in particular, the solder wicking along the multi-filament affects the durability of the joint and added stiffness to the textile. The distinct effect of the solder especially has an impact on the stiffness of the textile. All work steps were performed manually and were not fast enough. 

Additionally, the soldering of smart textiles was investigated in different public-funded German research projects. In these projects, conductors with and without insulation were used. The temperature sensors and LEDs could be soldered to the exposed conductors. The working steps were performed manually with a solder piston and automatically with a soldering robot, but tensioning and feeding of the strips were still done manually. Overall, the contacting processes, manually and automatically, were still too slow, and the conductors were not stretchable [22,23].

## 2. Materials and Methods

### 2.1. Different Types of Lasers

**Figure 2:**
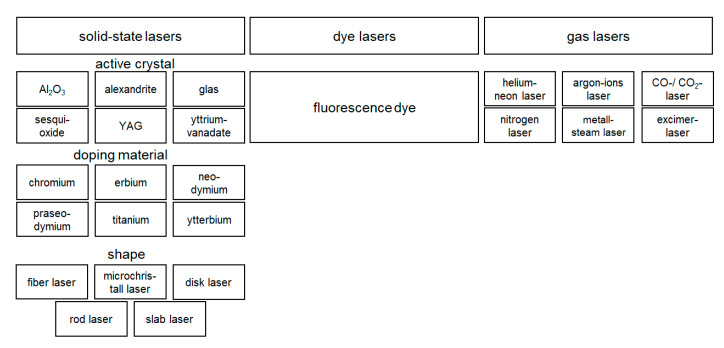
Characterization of different lasers using their active medium: solid-state lasers, color lasers, and gas lasers. Solid-state lasers can be distinguished by their active crystal, their doping material, and their shape.

Lasers can be distinguished according to their laser-active material and their pumping. The laser-active media can be divided into three classes: gas lasers, dye lasers, and solid-state lasers. These are pumped either optically by light or electrically by gas discharge, electrical discharge, or a laser diode. The laser-active medium of solid-state lasers depends on the active crystal used, the doping material, and its shape (see Figure 2). In dye lasers, a fluorescent dye is used as the active medium. Various gases are available for the construction of a gas laser.

The interaction of a laser-active medium and a resonator produces a monochromatic, coherent light beam with a very small divergence. The laser-active medium determines the wavelength of the laser beam and is able to influence the temporal behavior of the laser. The laser resonator determines other properties of the emitted laser radiation, such as beam divergence, beam diameter, intensity distribution, and pulse duration [24].

Active media with wavelengths between 940–1064 nm are used for laser soldering, since the ab-sorption coefficient of metals at these wavelengths is relatively high, and thus sufficient energy can be introduced into the material. The wavelength of Nd:YAG lasers is 1064 nm.

### 2.2. Solder Materials and Laser Soldering with Nd:YAG Fiber Lasers 1064 nm to Connect E-Textiles

The integration of PCB on textiles is usually divided into three process steps. First, the textile integrated wires have to be stripped. Afterward, the sensors/actuators can be contacted to these points. In a final work step, the applied sensors are protected from environmental influences and mechanical loads in the injection molding process. The use of a laser soldering process for the production of e-textiles is suitable for various reasons. On the one hand, the first two process steps in the production of e-textiles can be carried out in one machine bed; on the other hand, the laser soldering process ensures low heat input into the component [7]. Contactless soldering with high energy density enables short process times and low mechanical stress. This prevents damage to the surrounding textile. In addition, hard-to-reach areas can also be reached.

For laser soldering with solder paste, pastes with a grain size of 4 or 5 or larger should always be used. Smaller particles (larger grain size) have a larger surface to volume ratio. This promotes heat conduction between the particles. Heat conduction between the particles is important, as the laser energy can only be introduced optically via the surface. This means that the solder paste can only be melted by heat conduction. However, an enlarged surface also leads to increased oxide formation before and during the process.

**Figure 3:**
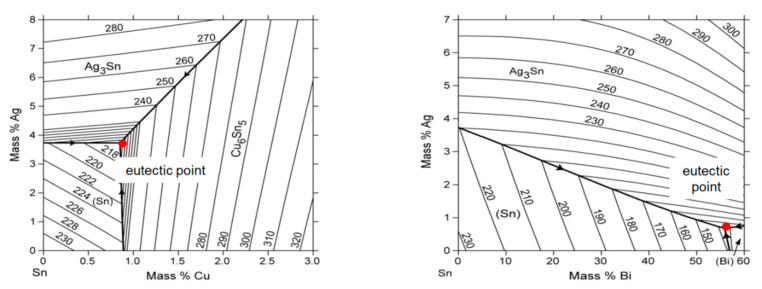
Phase diagram of SAC305 (**left**) [24] and Sn-Bi-Ag (**right**) [27] with eutectic points Sn96.5-Ag3-Cu0.5 (**right**) and Sn42-Bi57.6-Ag0.4 (**left**).

Two different alloys in a near-eutectic composition were used as a solder paste. As a result, the alloys have a fixed melting point so that a rapid solid-liquid transition (phase diagram) takes place. This is elementary for a stable process and for determining the laser parameters. In one attempt popular in electronics manufacturing, Sn96.5-Ag3-Cu0.5 (SAC305) with eutectic point at Sn96.5-Ag3.8-Cu0.7 (SAC 387) and a melting temperature of 216 °C was used [24]. SAC alloys are characterized by very low electrical resistance and low melting points as well as high reliability in contacting. SAC 305 is more recommendable than the eutectic SAC387 because it has a lower tendency to solder defects [25]. In another attempt, the alloy Sn42-Bi57.6-Ag0,4 with a eutectic point at 43.47Sn-55.85Bi-0.68Ag and a melting temperature of 137.1 °C was applied [26]. We have chosen these two alloys because of their relevance to electronics manufacturing processes. Both alloys have a blend ratio close to the eutectic point, so that they have a rapid solid to liquid transition. Figure 3 shows the phase diagrams of the two alloys and their proximity to the eutectic point.

### 2.3. Wire Stripping

The laser (LPKF Laser & Electronics MicroLine 3D 160i, Garbsen, Germany) movement layout for stripping the micro cable is shown in Table 1. The red crossed lines in this frame represent the movement paths of the laser focal point. The distance between two lines counts only 45 µm. In order to completely remove the outer housing of the micro cables during stripping, the laser processing must be repeated. The optimum number of repetitions was set at 12, which leads to the best stripping result. The total process takes about 2.4 s.

The high transmittance (approximately 90%) of polyester means that only a very small proportion of the laser beam is absorbed by the polyester fibers [28].

### 2.4. Laser soldering of PCB to Textile

The hatch pattern of the test soldering surface reflects the movement pattern of the laser beam. The distance between the two lines of the hatch pattern is 45 μm, by a position accuracy of ±25 µm. The focus diameter of the laser beam is 80 μm (λ = 1064 µm) and a distance between Laser and PCB of 212 mm. The laser alignment and positioning are controlled by a camera. A range of 4 mm × 1.4 mm is traversed with this. This area is then processed for soldering with the parameters from the following Table 2. The parameters were selected on the basis of preliminary tests and varied by a suitable combination of parameters. Pneumatic clamping jaws are used to fix the textiles on the processing table. This means that the position of PCB and the textile can no longer change. The soldering paste is dosed by means of a robot. Also, 4–12 mg of solder paste is applied per soldering point.

Statistical Design of Experiments (DoE) is a methodology for the planning and statistical evaluation of test series. The DoE aims to obtain the most important influencing factors on the measurement results (outputs). The relationships between input and output can be determined and the influence of the input variables and their interactions on the output can be quantified. Statistical design of experiments has proven its worth for complex applications compared to other methods with multivariable data analysis. A full factor plan examines all combinations. The number of trials n_r_ is calculated accordingly as follows:

$$n=n_{l}^{n_{f}}$$

n_l_ represent the number of levels and n_f_ the number of factors.

### 2.5. Continuity Test

**Figure 4:**
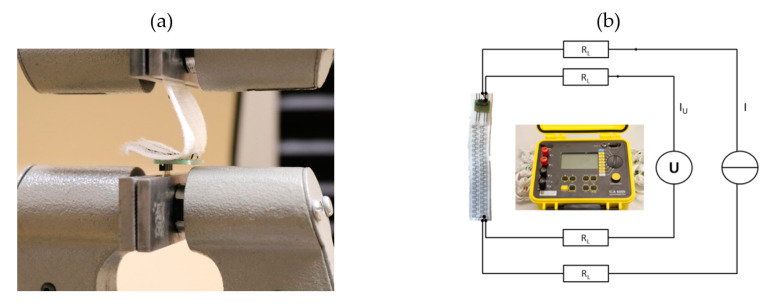
(**a**) Peeling test set-up: The PCBs are fixed in the blank and pulled on the contacted textile bands with a speed of 100 mm/min; (**b**) the measuring setup for determining the contact resistances by using the four-wire measurement technology.

The measurement of the contact resistance is an indicator of the quality of the contact itself. For a reliable measurement, a four-wire measuring instrument is used. It eliminates the line resistances of the measurement cables (Figure 4b). The falling voltage at the resistor is measured with a voltmeter over the two remaining conductors. Based on the ohmic law, the resistance of the contact is calculated. Contact resistances are in a range of only a few milliohms [17]. In order to generate a measurable voltage, high currents of up to 10 A are required.

### 2.6. Peeling Test

The peeling test is used to determine the mechanical strength of the joint for further processing. The force is applied perpendicular to the circuit board (Figure 4a). Therefore, a load cell with a maximum force of 1000 N is used. The travel speed is 100 mm/min.

## 3. Results and Discussion

### 3.1. Results of Wire Stripping

**Figure 5:**
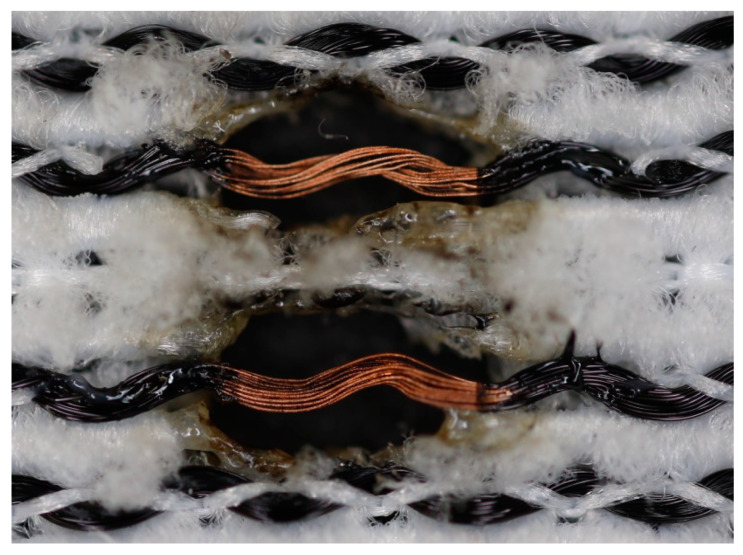
Laser wire stripping.

The textile-integrated lacquer-insulated copper strands were successfully and reliably stripped from both sides using the parameter set (12 repetitions, 16 W, 1200 mm/s, 85 kHz) and the hatch pattern (Table 1). The Nd:YAG laser (LPKF MicroLine 3D 160i with a wavelength of 1064 nm) is suitable for processing. This allows the electronics to be integrated locally into a textile tape without cutting off the integrated conductors. The insulation literally flaked off due to the high thermal energy of the laser (cf. Figure 5). The surrounding polyester knitted fabric was melted so that the copper strand could be contacted. It was still possible to find remains of the insulation on the conductors during microscopic examination of the stripped copper strands. Parts of the strands were always in the shadow of the laser beam, due to the 17-wire structure of the strands. That is why they probably were not removed.

### 3.2. Results of Laser Doldering

After laser stripping, the PCBs can be soldered directly to the textile conductor tape. The direct processing of the samples eliminates the need for a further positioning step. This promotes the automated integration of electronics on textiles. Two established alloys (Sn96.5-Ag3-Cu0.5 and 43.47Sn-55.85Bi-0.68Ag) from electronics manufacturing were selected for the soldering pastes. For both alloys, a parameter set was first empirically determined, and then a Design of Experiment was carried out. Since there are 27 parameter combinations per material, only a few good examples are listed here (Table 3). An initial assessment of the successful contacting was provided by the visual inspection. The pictures show the resulting contact points after laser soldering.

Table 3 shows that the parameter set 7 W, 95 mm/s and 100 kHz produces the best optical result. The applied solder paste is completely melted and there is no solder ball formation. None of the appearances can be directly assigned to a parameter. However, it is noticeable that the amount of energy is always in a similar range.

The optical evaluation on the basis of shape, surface, and traces of smoke of the soldering results from Table 4 leads to an optimal parameter set of 4 W, 100 mm/s, and 105 kHz for the solder paste 43.47Sn-55.85Bi-0.68Ag. In general, solder balls are formed in all samples with this solder paste. This could not be prevented even with the DoE. As a result, the SAC 305 alloy shows clear advantages in handling and results. After determining an optimum parameter set for the two alloys, SAC 305 is selected for further processing. The wires are stripped and soldered directly to the PCB. The solder quantity, the grain size of the SAC 305 alloys used, and the number of laser processes were varied.

The repeated melting of the solder paste has a positive influence on the soldering result (Table 5, No. 1 and 2). The renewed short-term melting of the solder paste improves the soldering result, because the solder has more time to flow into the strand, and therefore it has a better wetting behavior. The solder is not damaged by the repeated short term melting. From observing the surface of samples 1 and 3, it can be assumed that the heat distribution in the solder paste with smaller grains (T4) is better than in the paste with larger grains. This is caused by the same ratio of the higher surface to volume ratio. More contact points between the grains improve heat transfer in the solder paste, resulting in more uniform heating. In addition, we evaluated the mass used. This results in an optimum between 6 mg and 8 mg. The amount of solder used in pattern 1 is not sufficient, while pattern 7 contained clearly too much solder paste. The contacting quality was carried out in several tests, including the investigation of the contact resistances between conductor and pad surface. In visual inspection, the quality of solder connection can be analyzed very well. The most promising samples were examined in mechanical and electrical tests. The samples were produced with the parameter combination 4 from Table 5 (T4, 6.8, 3).

### 3.3. Continuity Test

The contact resistance provides information about the quality of the contact points. A four-wire measuring technique is used to calculate the contact resistance with the measuring setup from chapter 2.5. The contact resistance, as well as the wire resistance, can be calculated with the following formula:

R_ges_ = R_1+n_…R_L_ ≈ 2 R_K+n_∙R_L_ (2)

R_K_ = contact resistance; n = Number of wire (length 100 mm) and R_L_ = wire resistance

According to the data sheet, the line resistance is about 80 mΩ per conductor length (100 mm, AWG 32). Furthermore, the line resistance can be determined by measuring different conductor lengths.

**Figure 6:**
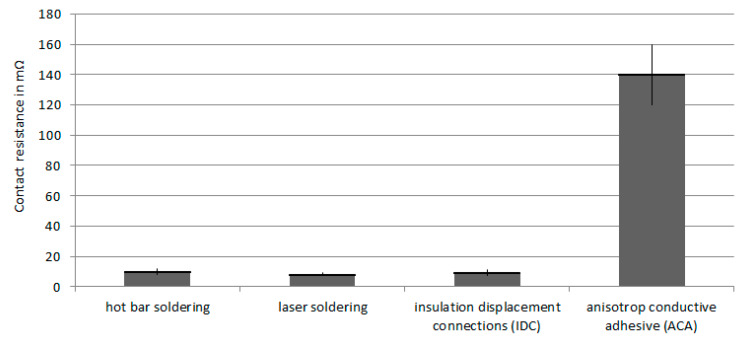
Contact resistance from hot bar, laser soldering, and insulation displacement connections (IDC). They all show nearly the same contact resistances, except for isotropic and anisotropic adhesive connections (ICA and ACA).

Figure 6 shows the nearly-identical contact resistances of the soldered joints and insulation dis-placement connections [7]. Aside from that, adhesive connections show a much higher contact resistance. At the same time, the line resistances from the data sheets can be confirmed by the measurement procedure.

### 3.4. Peeling Test

The inspection of the mechanical strength is also interesting, after the examination of the contact resistances. The bearable maximum force was determined in a peeling test. During the tensile test (Figure 4a), three types of failure were observed in particular:

(1) Pulling the micro cable out of the solder joint
(2) Break-off of the micro cable directly at the contact point
(3) Break-off the micro cable at the point up to which the strand draws tin during the soldering process

**Figure 7:**
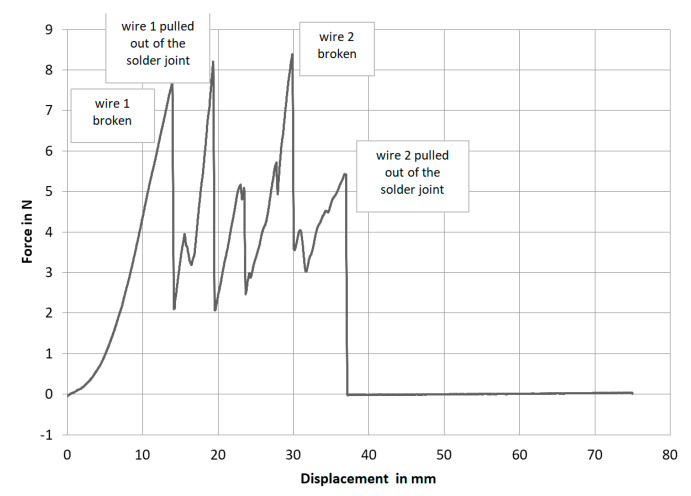
Exemplary force-displacement during the peel test.

**Figure 8:**
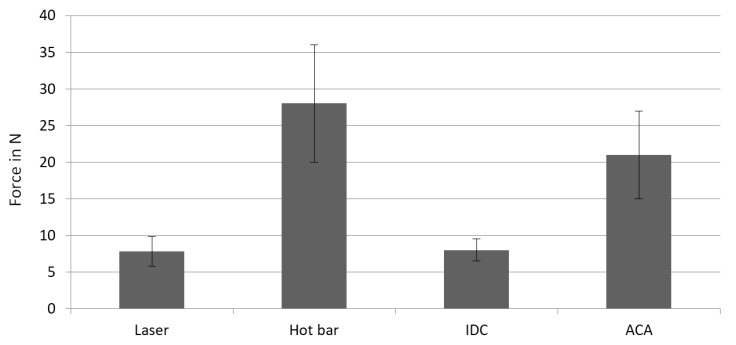
Peace force during peeling tests of laser soldering compared with the maximum peeling force of hot bar soldered, IDC, and ACA connections. Laser soldering shows a low peak force, with an acceptable scattering rate.

When peeling the laser-soldered samples, the following picture can always be seen. In the beginning the first strand breaks off at the soldering point. In the second step, the stranded wire breaks off the board completely. Then the applied force only acts on the second contacted strand until it breaks off. Finally, the second strand is also peeled off the board. This results in the characteristic curve shown in Figure 7 compared to the contacting processes investigated so far: hot bar soldering, insulation displacement connections (IDC), and anisotropic conductive adhesives (ACA). The laser-soldered connections have rather low mechanical strength (Figure 8) [7].

### 3.5. Metallography

**Figure 9:**
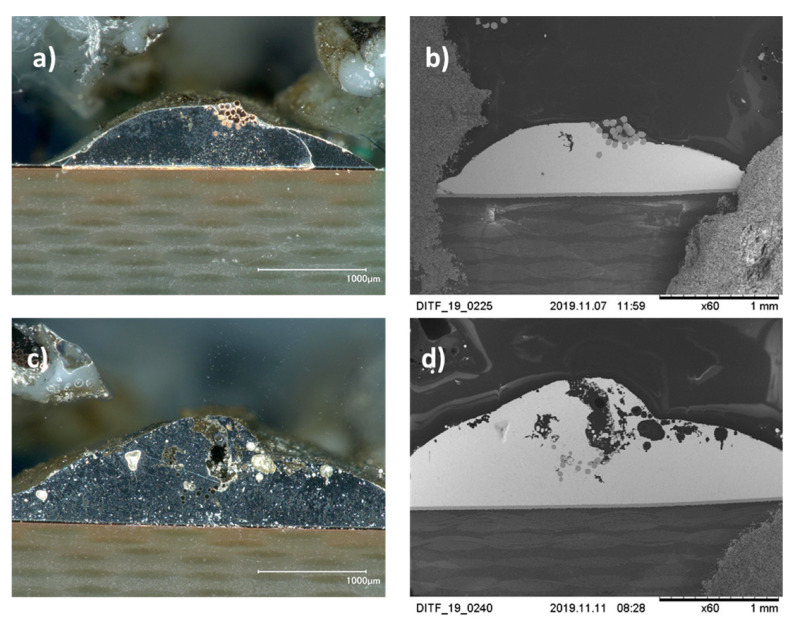
Pictures of the cross-sections of the soldered joints at 60× magnification; sample 1 (**a**,**b**) under OM (**a**) and SEM (**b**) with power 8W; sample 2 under OM (**c**) and SEM (**d**) with power 7 W. It can be seen that there is a blowhole at the solder joint of sample 2.

Metallography was performed to observe the microstructure of the solder joint. Two manufactured samples were used in this experiment, which were produced with different laser powers. To obtain the micrograph, an optical microscope (OM) and a scanning electron microscope (SEM) were used. The acceleration voltage of the SEM was 15 kV. Figure 9 shows the areas of the two samples 1 (a, b) and 2 (c, d) around the strands. Sample 1 was soldered with 8 W, and sample 2 was soldered with 7 W. It is obvious that there is a blowhole at the solder joint, c) and d). Noticeable is that not only a large blowhole in the solder joint is formed, but also many bubbles and pores. The numerous soldering defects indicate a poor mechanical connection of the soldered joint as well as low electrical conductivity. Sample 1 shows a bad wetting of the conductors.

**Figure 10:**
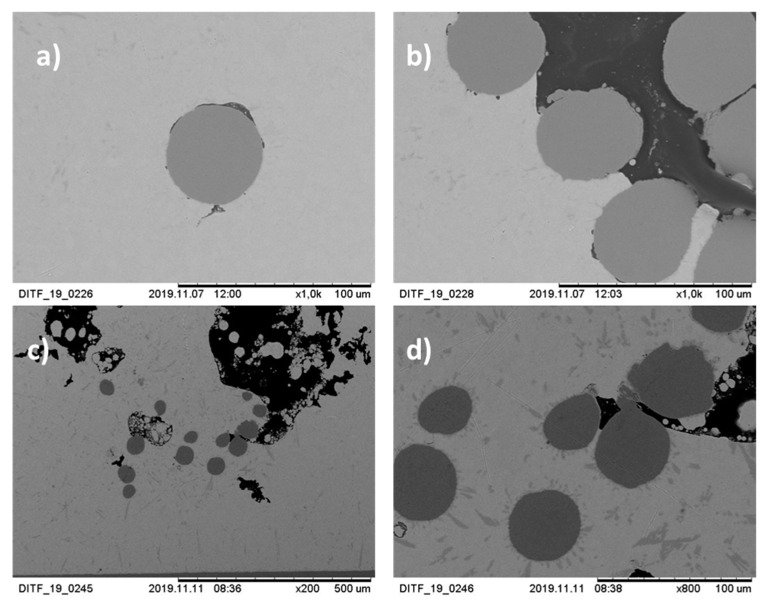
Picture of the cross-section of the solder joint at 200× to 1000× magnification: sample 1 under SEM at 1000× (**a**,**b**) magnification (power 8 W); sample 2 under SEM at 200× (**c**) and 800× (**d**) magnification (power 7 W).

In the following Figure 10, the cross-section of the specimens are shown at 200× to 1000× magnification. Many soldering defects can already be determined here. Cracks between the strands and the solder can be seen in sections a) and b). This leads to a reduction in strength between the strands and the solder. Figure 10c,d shows the plate-shaped structures. They promote local crack growth.

**Figure 11:**
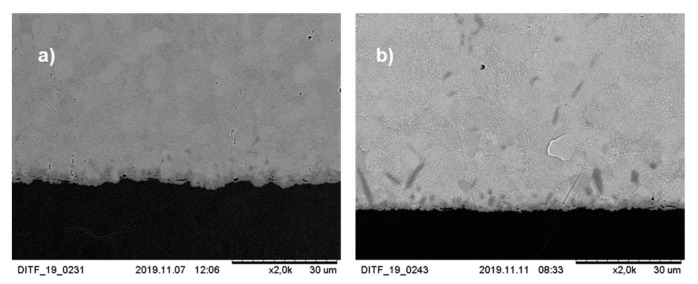
Pictures of the cross-section of the soldered joint with 2000× magnification: (**a**) power 8 W (**b**) power 7 W.

In addition, the Cu/SAC interface of the solder joint was observed under the scanning electron microscope with 2000-fold magnification. The composition of the intermetallic layers cannot be determined because no EDS mapping (energy-dispersive X-ray spectroscopy) was performed. An intermetallic layer can be seen in Figure 11. During soldering, Cu diffuses into the liquid SAC solder and reacts with Sn to form Cu_6_Sn_5_ at the Cu/SAC interface. Ag can react with Sn to form Ag_3_Sn. The structure of Cu_6_Sn_5_ is shell-shaped, while the structure of Ag_3_Sn is plate-shaped [29]. No plate-like structure is found in Figure 11a. In Figure 11b, it is visible that some plate-like structures are present at the interface.

## 4. Conclusions

The automated integration of electronics in textiles is still a major challenge for the production of smart textiles. In this paper, the feasibility of contacting electronics on textile-integrated insulated conductors by laser soldering was investigated. A pulsed Nd:YAG laser with a wavelength of 1064 µm was used as a laser source. It was demonstrated that integration is possible. Especially with lacquer-insulated conductors, this is a very elegant way of contacting, because the conductors can be stripped with the laser in one process step, and the electronic components can be contacted directly afterward. This makes a positioning step in the production process unnecessary. This is one of the main advantages of laser soldering electronics to textiles. For parameter determination, a full factorial test plan was carried out for contacting, and suitable parameters were determined. Unfortunately, however, in some cases there are poorly-wetted conductors. As a result, the mechanical strength as well as the electrical conductivity of a few contact points drop from the usual values for soldering.

## Figures and Tables

**Table 1:** Wire stripping parameter.

Repetitions	Laser Power (W)	Processing Speed (mm/s)	Frequency (kHz)	Laser Movement Layout
12	16	1200	85	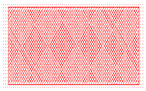

**Table 2:** Parameters of the full factor plan: soldering of PCB to textile with four different alloys: Sn96.5-Ag3-Cu0.5 (T3, T4), 43.47Sn-55.85Bi-0.68Ag (T3, T4).

Full Factor Plan: Alloy:	S(1)	S(2)	S(3)
Laser power (W) Sn96.5-Ag3-Cu0.5	6	7	8
Laser power (W) 43.47Sn-55.85Bi-0.68Ag	4	5	6
Processing speed (mm/s)	95	100	105
Frequency (kHz)	95	100	105

**Table 3:** Parameter and results with Sn96.5-Ag3-Cu0.5 (SAC 305).

43.47Sn-55.85Bi-0.68Ag				
No.	Laser Power (W)	Processing Speed (mm/s)	Frequency (kHz)	Results
1	6	100	95	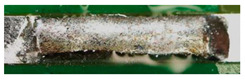
2	7	95	95	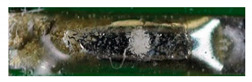
3	7	95	100	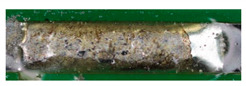
4	7	95	105	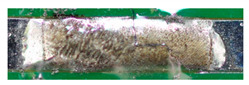
5	7	100	95	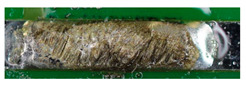
6	7	100	100	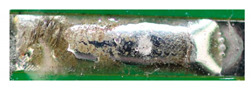
7	7	100	105	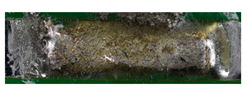
8	7	105	95	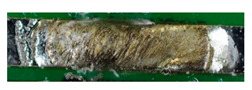
9	7	105	100	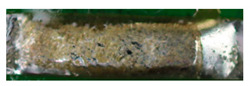

**Table 4:** Parameter and results for 43.47Sn-55.85Bi-0.68Ag.

43.47Sn-55.85Bi-0.68Ag				
No.	Laser Power (W)	Processing Speed (mm/s)	Frequency (kHz)	Results
1	4	95	105	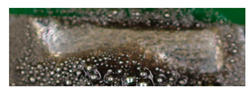
2	4	100	105	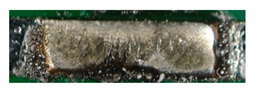
3	4	100	100	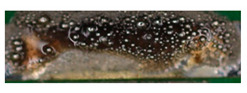
4	4	105	95	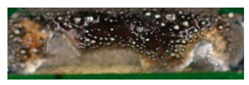
5	4	105	105	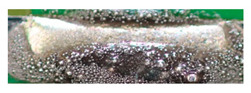
6	5	95	105	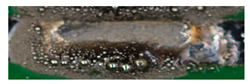
7	5	100	100	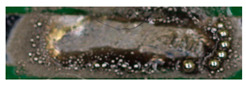
8	5	105	105	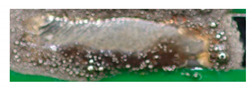

**Table 5:** Solder results depending on grain size and applied quantity.

No.	Grain Size	Solder (mg)	Laser Repetitions	Results
1	T3	3.4	3	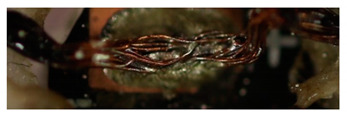
2	T3	3.4	4	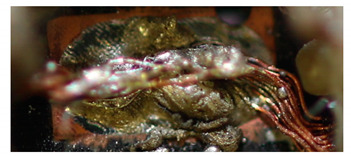
3	T4	4.1	5	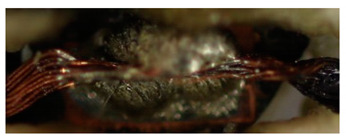
4	T4	6.8	3	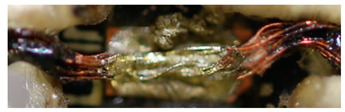
5	T4	8.7	2	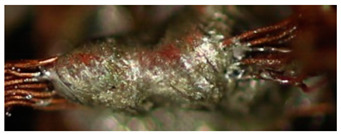
6	T4	11.1	5	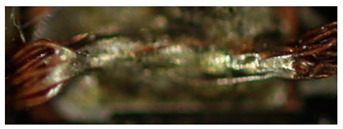
7	T4	19.9	2